# MRI-guided focused ultrasound for essential tremor

**DOI:** 10.3171/2024.7.FOCVID2472

**Published:** 2024-10-01

**Authors:** Alexander H. Agopyan-Miu, Grace B. Simmons, Gordon H. Baltuch

**Affiliations:** 1 Department of Neurological Surgery, Columbia University Medical Center, New York, New York; and; 2 Columbia University Vagelos College of Physicians and Surgeons, New York, New York

**Keywords:** MRI-guided focused ultrasound, essential tremor, operative technique

## Abstract

This video depicts a left-sided MRI-guided focused ultrasound (MRgFUS) thalamotomy for a right-arm tremor in a 76-year-old, right-handed man with bilateral upper-extremity tremors. The patient tolerated the procedure well, with immediate tremor reduction that persisted at the 1-month follow-up, and there were no adverse events noted during intraoperative testing, postoperative testing, or follow-up. This case highlights MRgFUS as an effective, safe, incision-free treatment option for medically refractory essential tremor. Additional research is required to establish the long-term efficacy of MRgFUS compared with other surgical treatment options such as deep brain stimulation (DBS) and radiosurgery.

The video can be found here: https://stream.cadmore.media/r10.3171/2024.7.FOCVID2472

## Transcript

This video demonstrates the benefit of MRI-guided focused ultrasound (MRgFUS) for the treatment of essential tremor.

### 0:26 Disclosure.

None of the authors have any conflicts of interest to disclose for this video. Informed consent to film and distribute this case was obtained from the patient the day of the procedure.

### 0:37 Essential Tremor.

Essential tremor is a chronic progressive syndrome primarily presenting as an action tremor involving the arms and hands. It is one of the most prevalent movement disorders worldwide, affecting approximately 25 million people of all ages globally.^1^ While essential tremor does not shorten life expectancy, it can lead to significant disability and impairments of quality of life by negatively impacting daily activities such as writing, eating, drinking, and grooming.^2,3^

### 1:03 Essential Tremor Treatment Overview.

Treatment for essential tremor is indicated for patients with symptoms that interfere with activities of daily living or quality of life, with the goal of symptom management. Pharmacotherapy with propranolol or primidone is first line. However, surgical options are indicated for appropriately selected patients that meet criteria for medically refractory tremor.^2,4^

### 1:24 Case Introduction.

As is the case for our 76-year-old, right-handed gentleman with bilateral upper-extremity tremors that have impacted his ability to independently feed and clothe himself. In addition to his upper-extremity tremors, he has been experiencing increased head and vocal tremors and gait instability. In light of inadequate tremor control with typical first-line medications, including primidone,^4^ this gentleman presented to our center for surgical management.

### 1:49 Surgical Treatment Options.

Multiple surgical options exist for the treatment of essential tremor, as listed here. Currently, deep brain stimulation is considered the gold standard for essential tremor given its established long-term safety compared to open thalamotomy.^5^ However, for patients opposed to implant placement or incisions, those with contraindications to open procedures, those who are unable to travel for frequent programming visits, or even those who are unable to receive a metallic implant, incision-free options like MRI-guided focused ultrasound or radiosurgery are enticing alternatives.^2^ While direct comparisons between MRI-guided focused ultrasound and DBS are still in their infancy, focused ultrasound may provide better postoperative quality of life improvement over DBS.^5,6^ Due to the relatively recent emergence of MRI-guided focused ultrasound, direct comparisons between it and radiosurgery have not clearly established one as a definitive incisionless treatment option.^7,8^ The ability to perform intraoperative testing and monitor for adverse events while using nonradiating energy to create more focal lesions with immediate effect are relative advantages of MRI-guided focused ultrasound over radiosurgery.^7,8^

### 2:58 Rationale for Procedure.

Our institution prefers a minimum skull thickness, typically expressed as a skull density ratio of at least 0.40, for MRI-guided focused ultrasound. We believe that skull density ratios above this threshold allow ultrasonic waves to effectively penetrate and heat the desired target, though this is not a strict threshold.^9^ Patients with skull density ratios of less than 0.40 can still be successfully treated with focused ultrasound. Since our patient had a favorable skull density ratio and was interested in an incision-free surgical approach, we elected for focused ultrasound to treat his essential tremor.

### 3:36 Frame Equipment.

Prior to the procedure the patient’s head must be fully shaved, as hair follicles interfere with ultrasound penetration. Premedication for the frame placement is provided as needed. This image here displays the typical supplies needed for frame placement. Local anesthetic and plastic screws are then used to affix the frame to the head of the patient.

### 3:56 Frame Application.

The frame should be placed as low as possible on the patient—just above the eyebrows anteriorly and at the level of the inion posteriorly—and it should be slightly offset toward the ablation target.

### 4:06 Silicone Application.

A circular silicone membrane is fitted around the patient and attached to the stereotactic frame, which is later attached to the focus ultrasound transducer on the MRI table. This membrane creates a watertight seal, which allows cooled, degassed water to circulate around the patient’s head to both cool the scalp during treatment and permit acoustic coupling between the ultrasound emitters and the head.

### 4:28 Silicone Application.

The patient is then brought into the MRI suite and laid supine on the MRI table. The stereotactic frame is locked to the ultrasound transducer on the MRI table.

### 4:38 Setting AC-PC.

A preablation MRI is obtained and registered to a preoperative CT. Next, AC-PC is defined on the MRI to assist with targeting.

### 4:47 VIM Targeting.

The ventral intermediate nucleus of the thalamus is defined by both direct visualization and with atlas coordinates that roughly correspond to being approximately 6–7 mm anterior to the posterior commissure and approximately 11 mm from the edge of the third ventricle at the level of the intercommissural plane. After target definition, confirm that the distance between the target and focal point of the transducer is < 2 mm. If not, mechanical movement of the transducer is made and another scan needs to be completed to ensure the proper distance between the transducer’s focal point and the target.

### 5:21 Defining No-Pass Regions.

No-pass regions are then defined in red. These correspond to areas that are at increased risk of ultrasound absorption and thus heating. For example, intracranial calcifications, sinuses, and choroid represent typical no-pass zones.

Finally, a check is made to ensure that enough transducer elements are active and distributed over a large enough area of skull to provide enough focused energy at the target to reach ablative temperatures. Generally, a greater number of elements allow us to deliver low energy across a larger surface of the skull, reducing scalp heating.

### 5:54 Treatment Alignment.

The procedure starts with a low-temperature heating at subtherapeutic temperatures (typically 40°C–45°C) to further adjust the focus on the desired target. Next, sonications are done at 45°C–50°C to assess the shape of the lesion and further adjust targeting as needed. Since these temperatures are still high enough to disrupt brain function, intraoperative tests can be performed to assess for adverse effects such as dysarthria, paresthesia, and paresis.

### 6:21 Ablating VIM.

At this point, sonications are then performed at 56°C–57°C, which is high enough to ablate tissue. These temperatures tend to create a 4- to 5-mm-diameter ablation at the target as evaluated on T2 sequences.^10^ During the entirety of ablative sonication, the shape of the lesion is monitored. Further, the acoustic spectrum and control panels adjacent to the temperature curves are monitored to ensure that treatments do not pass a threshold frequency where the risk of cavitation increases. Cavitation can lead to large local temperature fluctuations and/or undesirable mechanical effects on tissue.

### 6:58 Intraoperative Testing Part 1.

Again, one of the benefits of focused ultrasound over other incisionless alternatives is the ability to perform intraoperative testing, the results of which can be used to assess for adverse effects, and thus adjust targeting if needed as well as tremor suppression. Our entry procedure check-ins involved assessment of speech by recounting the days of the week, of stress by counting backward from 100, of adverse effects by asking the patient if they are experiencing any paresthesias, and of tremor suppression by drawing spirals and asking the patient to emulate drinking with an empty cup.

### 7:49 Intraoperative Testing Part 2.

As can be seen with each subsequent intraoperative spiral draw test, the patient’s tremor is increasingly suppressed.

Once an adequate lesion size and tremor suppression has been achieved, a final MRI is obtained to confirm the ablation target.

### 8:04 Postprocedure Testing.

Unlike thalamotomy with radiosurgery, the effects of MRI-guided focused ultrasound are immediate. The patient can reliably drink from a cup of water, and his writing is improved. In our center, patients are monitored in the recovery area for an hour prior to discharge home.

### 8:30 One-Month Follow-Up.

At their follow-up appointment, the patient denied any gait instability or numbness of his hands or lips. He reported excellent tremor suppression in his right hand as well as a reduction in his head tremor. He is interested in returning to our center for a right-sided thalamotomy to suppress his left upper-extremity tremor. He will have two more follow-up visits at 6 months and 1 year.

### 8:51 MRgFUS for ET Summary.

In summary, MRI-guided focused ultrasound is an effective, incision-free, same-day operation that can be used in the surgical treatment of medically refractory essential tremor. Unlike other incision-free modalities, MRI-guided focused ultrasound provides the most flexibility for intraoperative testing to ensure safe and effective lesion targeting. As part of the surgical armamentarium, MRI-guided focused ultrasound expands surgery to those with essential tremor who otherwise cannot or are not willing to undergo an open or more invasive procedure, thus serving as a safer, potentially equally efficacious alternative to other surgical options.^6^

## Acknowledgments

We thank the patient for allowing us to produce and disseminate their focused ultrasound session, Dr. Baltuch’s office staff, and the Cornell team who helped with patient care.

## Disclosures

The authors report no conflict of interest concerning the materials or methods used in this study or the findings specified in this publication.

## Author Contributions

Primary surgeon: Baltuch. Editing and drafting the video and abstract: all authors. Critically revising the work: all authors. Reviewed submitted version of the work: all authors. Approved the final version of the work on behalf of all authors: Agopyan-Miu. Supervision: Baltuch.

## Supplemental Information

### Patient Informed Consent

The necessary patient informed consent was obtained in this study.
